# Standardized voluntary force measurement in a lower extremity rehabilitation robot

**DOI:** 10.1186/1743-0003-5-23

**Published:** 2008-10-28

**Authors:** Marc Bolliger, Raphael Banz, Volker Dietz, Lars Lünenburger

**Affiliations:** 1 Spinal Cord Injury Center, Balgrist University Hospital, Zurich, Switzerland; 2 Sensory-Motor Systems Laboratory, ETH Zurich, Switzerland

## Abstract

**Background:**

Isometric force measurements in the lower extremity are widely used in rehabilitation of subjects with neurological movement disorders (NMD) because walking ability has been shown to be related to muscle strength. Therefore muscle strength measurements can be used to monitor and control the effects of training programs. A new method to assess isometric muscle force was implemented in the driven gait orthosis (DGO) Lokomat. To evaluate the capabilities of this new measurement method, inter- and intra-rater reliability were assessed.

**Methods:**

Reliability was assessed in subjects with and without NMD. Subjects were tested twice on the same day by two different therapists to test inter-rater reliability and on two separate days by the same therapist to test intra-rater reliability.

**Results:**

Results showed fair to good reliability for the new measurement method to assess isometric muscle force of lower extremities. In subjects without NMD, intraclass correlation coefficients (ICC) for inter-rater reliability ranged from 0.72 to 0.97 and intra-rater reliability from 0.71 to 0.90. In subjects with NMD, ICC ranged from 0.66 to 0.97 for inter-rater and from 0.50 to 0.96 for intra-rater reliability.

**Conclusion:**

Inter- and intra- rater reliability of an assessment method for measuring maximal voluntary isometric muscle force of lower extremities was demonstrated. We suggest that this method is a valuable tool for documentation and controlling of the rehabilitation process in patients using a DGO.

## Background

Muscle force testing is a well established method of assessing muscle function in subjects with neurological movement disorder (NMD) [1,2], despite the fact that these tests are in generally not sensitive enough to assess the force of a single muscle. Isometric force measurements are widely used because walking ability has been shown to be related to muscle strength [3-6]. Therefore, monitoring of muscle force can be used to control the effects of rehabilitation treatments. Furthermore, in rehabilitation hospitals, manual muscle tests (e.g. Manual Muscle Test, ASIA Motor score, Medical Research Council, Lower Extremity Motor Score) are the most commonly used methods of documenting impaired muscle strength. However, these tests are based on subjective assessment, produce ordinal (not scalar) data, require comprehensive training of therapists, and have poor inter- and intra-rater reliability [7,8]. In addition, these tests are usually not sensitive to small or moderate changes in muscle strength [1,9].

Robotic gait training devices have gradually become established to treat individuals with a locomotor dysfunction, such as spinal cord injury (SCI), stroke and traumatic brain injury [10-13]. A widely used device is the driven gait orthosis (DGO) Lokomat (Hocoma AG, Volketswil, Switzerland). This DGO is equipped with force transducers to assess the activity of patients while walking with the DGO. A detailed description of the Lokomat is published elsewhere [14,15]. Recently a novel measurement method for assessing muscle force using this DGO was developed. The method can be applied during a standard Lokomat training session and requires minimal additional time. The mechanical properties of the device allow hip and knee flexion and extension measurements.

The aim of this study was to analyze the reliability of a measurement method that assesses voluntary isometric force of leg muscles with a driven gait orthosis. We determined inter- and intra-rater reliability of force measurements in subjects with and without NMD. If reliability can be demonstrated, the new assessment method can be established as a tool to investigate and control the rehabilitation process of patients.

## Methods

### Isometric force measurement with the DGO

**Figure 1:**
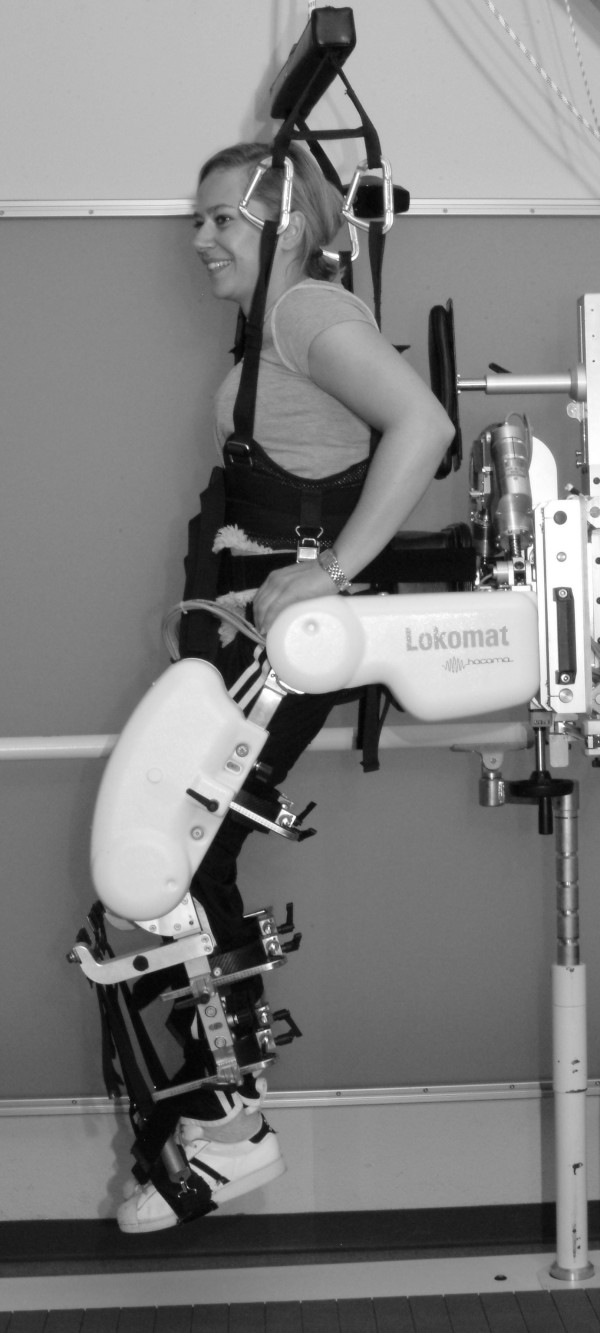
**Measuring position of subject in DGO**. Subject in the position used for the force measurement in the DGO. The device is set to position control mode with preset fixed joint angles (hip 30° flexion, knee 45° flexion).

The DGO Lokomat is used in combination with a treadmill and a dynamic body weight support system. The DGO controls the patient's leg trajectories in the sagittal plane during walking [14,15]. The hip and knee joints of the DGO are actuated by linear back-drivable actuators integrated into an exoskeleton structure. In every actuator, a force transducer measures the linear forces, whereas potentiometers measure the actual joint angles. The torques acting on each joint are calculated online from these position and linear force values based on the known geometry. For the isometric force assessment, subjects wear a harness and are fixed to the DGO by straps around the trunk and the pelvis. The legs of the device are attached to the subject's legs with cuffs around the thighs and calves. Proximal and distal leg structures of the DGO are adjusted to align hip and knee joints of the subjects with the joint axes of the DGO. Subjects are lifted above the treadmill (unloading from 100% body weight) and the software sets the device to position control mode with preset fixed joint angles (hip 30° flexion, knee 45° flexion; see Figure 1). In this position subjects are asked to perform either a flexion or extension movement in hip or knee joint in left or right leg and push against the orthosis legs according to a defined sequence of tests. The system controls the drives to keep this position and measures forces acting on the force transducers.

**Figure 2:**
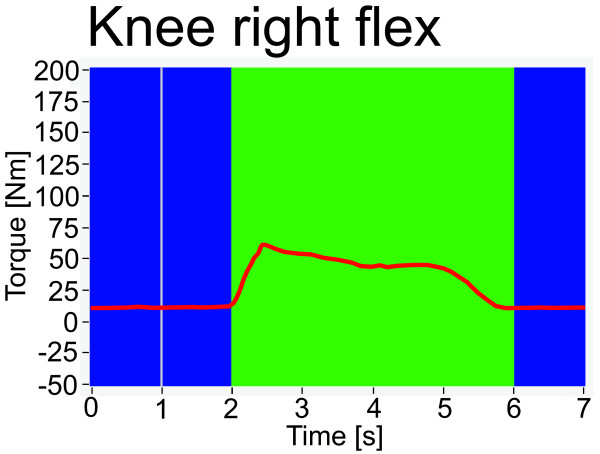
**Feedback display presented to subjects**. Display presented to the subjects during isometric muscle force tests. The curve represents the torque applied by the subjects to the DGO.

Visual feedback of the forces applied to the DGO is displayed for the subjects (Figure 2). Forces applied in the desired movement direction for the respective test led to an increase of the curve. However, subjects are not provided with knowledge about their absolute results (numeric torque values). Continuous data of angular position and torque are recorded and stored. As the result of each test, the maximal torque in a 5000 ms interval after the start cue is calculated using a moving average (width 1000 ms). A possible torque offset at test start is corrected by subtraction of the average torque between 2000 ms and 1000 ms before the start cue. Furthermore, subjects are instructed to be completely passive before the start cue. This method of calculating torque was used in the present study and is implemented in the commercially available DGO.

### Participants

The study protocol was approved by the local Ethics committee and conformed to the Declaration of Helsinki. All participants gave written informed consent before data collection. Sixteen subjects without neurological deficits (mean age 25.7, SD 3.8 years; all women) and fourteen subjects with NMD (mean age 53.5, SD 16.5 years; 6 women, 8 men) participated in the study. All subjects with NMD were able to understand and follow the instructions. Clinical diagnoses of subjects with NMD are shown in Table 1. Healthy athletic male volunteers were able to push the device markedly away from the desired position because the Lokomat's drives were not capable of maintaining position if very high forces were applied. Therefore measurements with subjects without NMD were accomplished only with female subjects expecting that they were not able to push the Lokomat away from the desired position.

**Table 1:** Characteristics of subjects with neurological gait disorder.

Subject No.	Sex	Age [years]	Type of Injury	Time post lesion [month]
P1	m	72.9	Stroke	1.7
P2	m	81.1	Stroke	0.8
P3	f	64.3	Stroke	36.1
P4	m	46.4	Intracranial Hemorrhage	48.1
P5	m	70.8	Stroke	4.0
P6	f	67.7	Brain tumor	4.0
P7	f	43.7	Stroke	1.5
P8	m	31.5	TBI	60.2
P9	f	45.2	Hypoxia	140.3
P10	f	26.2	CP	314.8
P11	f	35.0	TBI	155.3
P12	m	55.1	Stroke	1.6
P13	m	53.7	Guillain-Barré syndrome	6.6
P14	m	55.6	SCI	1.6

Abbreviations: *TBI *Traumatic Brain Injury; *CP *Cerebral Palsy; *SCI *Spinal Cord Injury

### Procedures

We assessed maximal voluntary isometric (static) contraction in subjects with and without NMD. Four muscle groups were tested: hip flexors, hip extensors, knee flexors and knee extensors for the right and left leg respectively. Subjects were tested independently by two experienced raters (rater A, rater B) on the same day to determine inter-rater reliability. Additionally, retests were conducted on the following day by rater A to access intra-rater reliability. Both raters were very experienced users of the DGO. The order of testing by rater A and rater B on day 1 was randomized to reduce the effects of subject bias, which could be caused by a learning effect or fatigue. Each rater was blinded to the results obtained by the other rater. For the measurements on day 1, subjects were fixed into the Lokomat by the first rater and were then familiarized with the testing protocol (at least two repetitions). After the familiarization, subjects had a break to relax and then performed two tests (trials) with the first rater. Afterwards they were taken out of the Lokomat and had a break of at least 2 minutes to avoid muscle fatigue during the tests by the second rater. After subjects reported recovery, the second rater fixed them into the Lokomat and performed another two tests (trials). For the measurement on day 2, subjects were fixed in the device by rater A and then performed 2 tests (trials) with a resting period of at least 2 minutes in between. For each force test the command "3-2-1-go" was used to initiate the measurement. The command was displayed on a computer screen and additionally given verbally by the rater. Subjects were instructed to produce force as fast and as hard as possible after the "go" signal and were required to hold maximum force during at least 3 seconds.

Additionally a single case study of one subject with an acute incomplete spinal cord injury (ASIA B, Th 12, 6 weeks post injury) was accomplished. Over the period of a 10 week training program with 3 DGO training sessions per week, force measurements on the DGO were conducted every 7–10 days and compared with walking tests (Timed Up & Go, 10-meter walk test, 6-minute walk test), which were assessed in the same time frame. Lokomat training sessions lasted 60 minutes and included at least 30 minutes of walking.

### Statistical analysis

We evaluated reliability using analysis of variance (ANOVA)-based intraclass correlation coefficients (ICC). ICCs were calculated with SPSS (SPSS 14 for Windows, release 14.0.0, SPSS Inc., Chicago, IL, USA). To test reliability for subjects with and without NMD, we calculated ICCs (2-way random-effects model) by using both single values (in each case the first measurement of rater A and rater B) and average values (average of the 2 measurements for every joint and every direction). ICC scores were compared with the following scale for interpretation of correlation: good (1.00 – 0.8), fair (0.80 – 0.60), and poor (< 0.60) [16]. ICC > 0.80 has been suggested to be feasible for clinical work but also ICC between 0.60 and 0.80 can provide researchers with valuable information [16]. Additionally standard error of measurement (SEM) and coefficient of variation of the method error (CV_ME_) were calculated. While the ICC reflects the degree of consistency of a measurement and is unit free, the SEM provides information about the expected trial-to-trial noise in the measured data and has the same units as the measurement of interest [17]. CV_ME _reflects the percentage difference of the measured parameter from test to test. Because this statistical result is unit free, it allows for a comparison across different studies [18]. In control subjects without NMD, we calculated reliability for the right and the left leg separately. In subjects with NMD, we calculated reliability for the more affected and the less affected side independently.

## Results

In subjects without neurological gait disorders, a total of 768 force measurements, 96 for each joint and movement direction, were acquired to assess inter- and intra rater reliability. The results showed fair to good inter- and intra-rater reliability for ICCs calculated from single as well as from averaged measurements (see Table 2, which shows results for ICCs, SEMs and CV_ME_).

**Table 2:** Inter-and intrarater reliability for subjects without neurological movement disorders (ICC 2,1-formula).

	Interrater						Intrarater					
		
	single measurement			average measurement			single measurement			average measurement		
		
Joint	ICC	SEM [Nm]	*CV*_ME _[%]	ICC	SEM [Nm]	*CV*_ME _[%]	ICC	SEM [Nm]	*CV*_ME _[%]	ICC	SEM [Nm]	*CV*_ME _[%]
***right side***												
hip flexion	0.92	6.2	8	0.95	4.4	5	0.83	8.3	11	0.89	6.6	9
hip extension	0.95	5.5	5	0.95	5.2	6	0.90	7.3	8	0.87	8.8	11
knee flexion	0.85	5.4	10	0.97	2.2	4	0.86	5.0	9	0.85	5.2	10
knee extension	0.92	5.0	7	0.96	3.4	5	0.71	8.9	8	0.90	5.2	7
***left side***												
hip flexion	0.84	9.8	13	0.97	4.0	5	0.76	9.9	12	0.75	10.5	14
hip extension	0.72	10.3	13	0.91	5.5	7	0.82	8.1	10	0.74	9.6	12
knee flexion	0.89	3.9	8	0.93	2.9	6	0.86	3.9	11	0.81	4.7	9
knee extension	0.91	5.8	8	0.97	3.4	5	0.89	6.2	10	0.89	6.0	9

Abbreviations: *ICC*, Intraclass correlation coefficient; *SEM*, standard error of measurement; *CV*_ME_, coefficient of variation of method error

In volunteers with neurological gait disorders, 672 measurements were collected, 84 for each joint and movement direction, to assess inter- and intra rater reliability. ICC for inter-rater reliability ranged from 0.66 to 0.97 and from 0.50 to 0.91 for intra-rater reliability. Reliability was fair to good for ICCs calculated from single as well as from averaged measurements. The exception was poor intra-rater reliability for hip flexion on the more-affected side if assessed with a single measurement. Detailed results (ICC, SEM and CV_ME_) are shown in Table 3.

**Table 3:** Inter-and intrarater reliability for subjects with neurological movement disorders (ICC 2,1-formula).

	Interrater						Intrarater					
		
	single measurement			average measurement			single measurement			average measurement		
		
Joint	ICC	SEM [Nm]	*CV*_ME _[%]	ICC	SEM [Nm]	*CV*_ME _[%]	ICC	SEM [Nm]	*CV*_ME _[%]	ICC	SEM [Nm]	*CV*_ME _[%]
***more affected side***												
hip flexion	0.86	6.8	11	0.90	6.2	10	0.50	11.6	26	0.79	7.6	17
hip extension	0.88	8.4	25	0.92	7.0	18	0.87	8.9	33	0.91	6.8	26
knee flexion	0.97	3.6	13	0.96	3.7	15	0.88	6.0	29	0.93	4.5	22
knee extension	0.96	4.5	9	0.85	7.9	12	0.86	8.4	22	0.86	8.2	21
***less affected side***												
hip flexion	0.90	7.1	10	0.96	4.4	7	0.78	10.0	19	0.82	8.8	16
hip extension	0.66	19.3	36	0.87	11.2	21	0.81	14.6	27	0.89	10.5	20
knee flexion	0.93	5.8	16	0.95	5.0	14	0.91	6.5	20	0.96	4.1	12
knee extension	0.86	8.2	17	0.92	5.5	11	0.85	7.1	14	0.84	7.0	14

Abbreviations: *ICC*, Intraclass correlation coefficient; *SEM*, standard error of measurement; *CV*_ME_, coefficient of variation of method error

**Figure 3:**
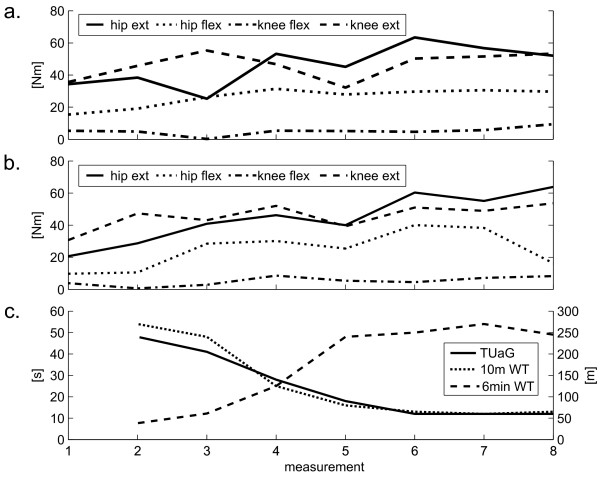
**Course of a patient's force measurements**. Isometric force measurements compared to walking tests assessed 8 times over a 10 week rehabilitation period of a single subject with acute incomplete spinal cord injury: a) maximal isometric force measurements of right leg (hip ext, hip extension; hip flex, hip flexion; knee flex, knee flexion; knee ext, knee extension), b) maximal isometric force measurements of left leg, c) walking tests (TUG, Timed Up & Go test, 10 m WT, 10-meter walk test; 6 min WT, 6-minute walk test).

The results of the single case study are shown in Figure 3. There is an indication that increasing isometric force of the patient is reflected in increasing performance in the walking tests.

## Discussion

The aim of this study was to evaluate inter- and intra-rater reliability of a recently developed measurement method assessing isometric muscle force in a driven gait orthosis (DGO). Therefore two experienced therapists tested 16 subjects without and 14 subjects with NMD on the same day to assess inter-rater reliability, and one therapist tested the subjects on two separate days to assess intra-rater reliability. Our results showed that the developed assessment tool for a DGO is a reliable tool for measuring isometric torques in subjects with and without neurological movement disorders. Therefore, it can be applied as an objective outcome measure in rehabilitation units. This novel method allows therapists to assess the muscle status of their patients walking in the DGO with a timesaving method and additionally to control and document the rehabilitation process.

Previous studies have established that isometric tests of muscular function show poor to good reliability depending on the device used to assess the muscle force. For instance, Scott et al. demonstrated for hip flexion and extension fair to good (0.65 – 0.87) inter-rater reliability assessed with a handheld dynamometer and poor to good (0.48 – 0.91) inter-rater reliability assessed with a portable dynamometer anchoring station [19]. Also a fair to good intra-rater reliability (0.76 – 0.98) for hip and knee flexion and extension movement with a slightly lower inter-rater reliability (0.64 – 0.97) was reported using a strain gauge [2]. Using isokinetic dynamometry to measure isometric muscle force mainly results in good reliability. Quittan et al. [20] showed ICC values for intra-rater reliability for knee flexion and extension between 0.82 and 0.99.

A direct comparison of our results with the above mentioned studies is not possible since we assessed isometric muscle force under different conditions. While subjects in the other studies were in a seated or recumbent position during strength testing, our subjects were in an upright position, mounted to the DGO and suspended with their whole body weight. However, we could also show fair to good inter- as well as intra-rater reliability for our voluntary isometric force measurements. Reliability was slightly higher in subjects without NMD. In contrast with the results of Meldrum et al. [2] inter-rater reliability was somewhat higher than intra-rater in both groups. This might have been due to the fact that measurements for testing inter-rater reliability were performed on the same day, whereas those for testing intra-rater reliability were conducted on two different days. To produce repeatedly maximal isometric force, a high motivation and full concentration are required from the tested subject [21]. This might have been difficult for some subjects and motivation might have differed on the two testing days. We were not able to control these subject-dependent factors. An additional reason for the lower intra-rater reliability could also have been that some subjects reported aching muscles from the force measurements on day one. This could have been resulted in somewhat poorer performance on day two and consequently resulted in lower intra-rater reliability. A longer break of 3 to 5 days between the two measurements might have reduced this effect.

In subjects without NMD 5 of 32 ICC values showed fair and the rest good reliability. In subjects with NMD 3 of 32 values showed fair, 28 values good reliability and one ICC value was below 0.6. This single poor reliability coefficient increased markedly when the average of two force measurements was used to calculate reliability. This goes in line with another study that suggested that more repetitions in a testing protocol might lead to better reliability [21]. The results from subjects with NMD supported this suggestion in most instances. Intraclass correlation coefficients (ICC) calculated from averaged measurements of the two successive trials were in the majority of cases higher than ICC assessed from single measurements.

The lower reliability values for hip force measurements compared to the knee force measurements indicate that performing a hip extension or flexion movement is more difficult than the knee task. This observation agrees with the results from Meldrum et al. [2] who also observed lower inter- and intra-rater reliability in hip compared to knee extension and flexion measurements.

The relative variation of the measurement error (CV_ME_) in subjects without NMD was low for inter- and intra-rater reliability (7 – 14%). This shows that the method will be capable of detecting small changes in isometric muscle force. CV_ME _were higher in the group of subjects with NMD (9 – 36% for single measurements and 7 – 26% for averaged measurements).

Even if these values seem to be large, the new method would have detected the changes of a 16 to 24 week training study by Cramp et al. in subjects with unilateral stroke 6 – 12 month post onset where an increase of 58% in isometric torque production in knee extensor muscle group was found [6]. Also the improvement of 29% in isometric knee extensor force in subjects with chronic incomplete spinal cord injury after a 12 week resistance training [22] would have been detected by the measurement method.

The large heterogeneity in the group of subjects with NMD was chosen because our goal was not to establish reliability values for a specific subject group but rather to investigate if the method is applicable to a wide range of subjects with NMD due to different etiologies. Nevertheless we expect better reliability for a more homogeneous subject groups.

Although reliability was slightly lower when using single measurements than using the average of two measurements, measurements with a single trial match best with clinical daily practice. In a clinical setting, tests are required that deliver reliable data with a minimum of time expenditure. With the presented method, therapists can assess voluntary muscle force during a training session in the DGO and reliably monitor the course of voluntary force generation in leg muscles. Regardless, in cases that require highly reliable force measurements, we propose performing two consecutive measurements in order to minimize bias and enhance reliability.

The fact that healthy athletic male subjects were able to push the DGO out of the desired position limits the application area of the method. Nevertheless we propose the method as appropriate for subjects being trained in the DGO. These subjects are generally very weak or in the case of subjects with hemiparesis the focus of therapists lies on the weak and affected side. The method was developed to optimize the monitoring of the rehabilitation process of subjects training in the DGO. As soon as subjects become too strong for DGO trainings, muscle force measurements have to be assessed with a different device, as necessary.

The ability of the method to document the rehabilitation process is shown in Figure 3. Increasing force measurements go along with increasing outcome measures. Whereas at time point 1 no clinical outcome measures could be collected because the subject was too weak to walk (even with assistance), DGO training and consequently muscle force measurements in the DGO were possible. Additionally it appears that the change in clinical gait function could be more related to changes in extensor muscles (hip and knee) than to those in flexor muscles. This goes in line with the observation that hip and knee extensors are the basic determinant for limb stability during stance phase [23]. Also Cramp et al. [6] reported that after a low intensity strength training in chronic stroke patients knee extensor force increased significant and correlated with gait speed while knee flexors did not change significantly.

Our preliminary data show the potential of the tool to document and control the rehabilitation process of subjects being trained in the DGO Lokomat. Future studies will be needed to investigate this observation.

## Conclusion

The assessment of maximal voluntary muscle force of hip flexors and extensors, as well as knee flexors and extensors in patients with NMD by the DGO Lokomat, produced reliable results. Intra-rater reliability was lower than inter-rater reliability. There was an increase in reliability when the average of the two trials was used to calculate ICC in comparison to when only one measurement was used. The presented assessment method might represent a valuable tool to document the course of rehabilitation in subjects with NMD.

## Abbreviations

*NMD*: Neurological movement disorders; *DGO*: Driven gait orthosis; *ICC*: Intraclass correlation coefficient; *SCI*: Spinal cord injury; *TBI*: Traumatic brain injury; *CP*: Cerebral palsy; *ME*: Method Error; *CV*_*ME*_: Coefficient of variation of the method error; *SEM*: Standard error of measurement.

## Competing interests

MB and LL were employed by the University of Zurich via a CTI (Commission for Technology and Innovation) project funded by the Swiss Bureau of Education and Technology and Hocoma AG, Volketswil, Switzerland, the producer of the Lokomat. Today, LL is employed by Hocoma AG, Volketswil, Switzerland, the producer of the Lokomat. RB was employed by the University of Zurich with funding from Hocoma AG, Volketswil, Switzerland. VD is Director of the Spinal Cord Injury Center of the University Hospital Balgrist and Professor for Paraplegiology at the University of Zürich, Switzerland.

## Authors' contributions

MB developed the study design and the software, performed data acquisition, completed the data analysis, and wrote the manuscript. RB aided in the study design, and in the data acquisition as well as in revising the manuscript. VD provided expert guidance on experimental design, assisted with data interpretation, and edited the manuscript. LL provided expert guidance on experimental design, developed the software, assisted with data interpretation, and edited the manuscript.

## References

[B1] Li RC, Jasiewicz JM, Middleton J, Condie P, Barriskill A, Hebnes H, Purcell B (2006). The development, validity, and reliability of a manual muscle testing device with integrated limb position sensors. Arch Phys Med Rehabil.

[B2] Meldrum D, Cahalane E, Keogan F, Hardiman O (2003). Maximum voluntary isometric contraction: investigation of reliability and learning effect. Amyotroph Lateral Scler Other Motor Neuron Disord.

[B3] Kim CM, Eng JJ, Whittaker MW (2004). Level walking and ambulatory capacity in persons with incomplete spinal cord injury: relationship with muscle strength. Spinal Cord.

[B4] Marino RJ, Graves DE (2004). Metric properties of the ASIA motor score: subscales improve correlation with functional activities. Arch Phys Med Rehabil.

[B5] Kim CM, Eng JJ (2003). The relationship of lower-extremity muscle torque to locomotor performance in people with stroke. Phys Ther.

[B6] Cramp MC, Greenwood RJ, Gill M, Rothwell JC, Scott OM (2006). Low intensity strength training for ambulatory stroke patients. Disabil Rehabil.

[B7] Bohannon RW (2005). Manual muscle testing: does it meet the standards of an adequate screening test?. Clin Rehabil.

[B8] Merlini L, Mazzone ES, Solari A, Morandi L (2002). Reliability of hand-held dynamometry in spinal muscular atrophy. Muscle Nerve.

[B9] Noreau L, Vachon J (1998). Comparison of three methods to assess muscular strength in individuals with spinal cord injury. Spinal Cord.

[B10] Wirz M, Zemon DH, Rupp R, Scheel A, Colombo G, Dietz V, Hornby TG (2005). Effectiveness of automated locomotor training in patients with chronic incomplete spinal cord injury: a multicenter trial. Arch Phys Med Rehabil.

[B11] Hesse S, Schmidt H, Werner C, Bardeleben A (2003). Upper and lower extremity robotic devices for rehabilitation and for studying motor control. Curr Opin Neurol.

[B12] Hornby TG, Zemon DH, Campbell D (2005). Robotic-assisted, body-weight-supported treadmill training in individuals following motor incomplete spinal cord injury. Phys Ther.

[B13] Husemann B, Muller F, Krewer C, Heller S, Koenig E (2007). Effects of locomotion training with assistance of a robot-driven gait orthosis in hemiparetic patients after stroke: a randomized controlled pilot study. Stroke.

[B14] Colombo G, Joerg M, Schreier R, Dietz V (2000). Treadmill training of paraplegic patients using a robotic orthosis. J Rehabil Res Dev.

[B15] Colombo G, Wirz M, Dietz V (2001). Driven gait orthosis for improvement of locomotor training in paraplegic patients. Spinal Cord.

[B16] Sleivert GG, Wenger HA (1994). Reliability of measuring isometric and isokinetic peak torque, rate of torque development, integrated electromyography, and tibial nerve conduction velocity. Arch Phys Med Rehabil.

[B17] Weir JP (2005). Quantifying test-retest reliability using the intraclass correlation coefficient and the SEM. J Strength Cond Res.

[B18] Portney LG, Watkins MP (2000). Statistical Measures of Reliability. Foundations of Clinical Research: Application to Practice.

[B19] Scott DA, Bond EQ, Sisto SA, Nadler SF (2004). The intra- and interrater reliability of hip muscle strength assessments using a handheld versus a portable dynamometer anchoring station. Arch Phys Med Rehabil.

[B20] Quittan M, Wiesinger GF, Crevenna R, Nuhr MJ, Sochor A, Pacher R, Fialka-Moser V (2001). Isokinetic strength testing in patients with chronic heart failure – a reliability study. Int J Sports Med.

[B21] Wilson GJ, Murphy AJ (1996). The use of isometric tests of muscular function in athletic assessment. Sports Med.

[B22] Gregory CM, Bowden MG, Jayaraman A, Shah P, Behrman A, Kautz SA, Vandenborne K (2007). Resistance training and locomotor recovery after incomplete spinal cord injury: a case series. Spinal Cord.

[B23] Perry J (1992). Gait analysis: Normal and pathological function.

